# Supplementary material for: Resonant thermal energy transfer to magnons in a ferromagnetic nanolayer
Source: Nat Commun. 2020 Aug 17;11:4130. doi: 10.1038/s41467-020-17635-1 (PMC7431562; doi:10.1038/s41467-020-17635-1)
Supplement: Supplementary file 1 — Supplementary Information [file 41467_2020_17635_MOESM1_ESM.pdf]

Resonant thermal energy transfer to magnons in a ferromagnetic nanolayer

Kobecki et al.

Supplementary Information

Supplementary Note 1. Modeling of heat transport for periodically pulsed optical excitation

In order to describe the heat transport in the system of a plane metallic film on a substrate we used the one-dimensional two-temperature model for the lattice heating temperatures of the film, T_{f}, and the substrate, T_{s}:

$$c_{\mathrm{f}}\frac{\partial T_{\mathrm{f}}}{\partial t}-\kappa_{\mathrm{f}}\frac{\partial^2 T_{\mathrm{f}}}{\partial z^2}=F_{\mathrm{p}}A_{\mathrm{f}}g(t)\frac{dS}{dz}, \tag{1}$$

$$c_{\mathrm{s}}\frac{\partial T_{\mathrm{s}}}{\partial t}-\kappa_{\mathrm{s}}\frac{\partial^2 T_{\mathrm{s}}}{\partial z^2}=F_{\mathrm{p}}A_{\mathrm{s}}g(t)\frac{dS}{dz}, \tag{2}$$

where c_j, κ_j, and A_j are the heat capacity, thermal conductivity, and fraction of absorbed optical energy relative to the incident excitation energy (further referred to as absorption coefficients), respectively ($j=\mathrm{f},\mathrm{s}$). The pump laser pulse fluence $F_{\mathrm{p}}=2W/(f_0\pi r^2)$, where W is the average laser power, $f_0=10$ GHz is the laser repetition rate, and r is the radius of the pump spot defined by $1/\mathrm{e}^2$ of the intensity. The function, $g(t)=\frac{2}{\tau_{\mathrm{p}}}\sqrt{\frac{2}{\pi}}\sum_{n=1}^{\infty}\exp\left(-2\frac{\left(t-\frac{n-1}{f_0}\right)^2}{\tau_{\mathrm{p}}^2}\right)$, describes the temporal evolution of the laser pulses with duration, τ_{p}, and repetition rate, f_0. Everywhere throughout the Supplementary Information, we chose the coordinate system with the direction of the z-axis pointing from the substrate to the film.

We found the absorption coefficients from the solution of Maxwell equations for the three-layer (air/metal/substrate) system:

$$A_{\mathrm{f}}=1-\left|\frac{(k_{\mathrm{a}}k_{\mathrm{f}}-k_{\mathrm{f}}k_{\mathrm{s}})\cos(k_{\mathrm{f}}h)-\mathrm{i}(k_{\mathrm{a}}k_{\mathrm{s}}-k_{\mathrm{f}}^2)\sin(k_{\mathrm{f}}h)}{(k_{\mathrm{a}}k_{\mathrm{f}}+k_{\mathrm{f}}k_{\mathrm{s}})\cos(k_{\mathrm{f}}h)-\mathrm{i}(k_{\mathrm{a}}k_{\mathrm{s}}+k_{\mathrm{f}}^2)\sin(k_{\mathrm{f}}h)}\right|^2-A_{\mathrm{s}}, \tag{3}$$

$$A_{\mathrm{s}}=\mathrm{Re}(n_{\mathrm{s}})\left|\frac{2k_{\mathrm{a}}k_{\mathrm{f}}}{(k_{\mathrm{a}}k_{\mathrm{f}}+k_{\mathrm{f}}k_{\mathrm{s}})\cos(k_{\mathrm{f}}h)-\mathrm{i}(k_{\mathrm{a}}k_{\mathrm{s}}+k_{\mathrm{f}}^2)\sin(k_{\mathrm{f}}h)}\right|^2, \tag{4}$$

where $k_j=k_jn_j$ is the complex wave vector of light in the air (j=a), film (j=f), and substrate(j=s), n_j is the complex refractive index, h is the thickness of the film.

The function $dS(z)/dz$ describes the spatial distribution of the energy density deposition by the pump light, which penetrates into the medium. More specifically, $S(z)$ is normalized to the value of the flux of electromagnetic energy at the metal surface. We used COMSOL Multiphysics® [1] to calculate $S(z)$.

The Supplementary equations 1-4 must be complemented by the boundary conditions. At the interface between the film and substrate (i.e. at $z=0$), the boundary conditions read:

[1]Experimentelle Physik 2, Technische Universität Dortmund, Otto-Hahn-Str. 4a, 44227 Dortmund, Germany. [2]Ioffe Institute, Politechnycheskaya 26, 194021 St. Petersburg, Russia. [3]Department of Theoretical Physics, V.E. Lashkaryov Institute of Semiconductor Physics, Pr. Nauky 41, 03028 Kyiv, Ukraine. [4] LAUM, CNRS UMR 6613, Le Mans Université, 72085 Le Mans, France. [5]School of Physics and Astronomy, University of Nottingham, Nottingham NG7 2RD, United Kingdom.
*e-mail: michal.kobecki@tu-dortmund.de; alexey.shcherbakov@tu-dortmund.de.

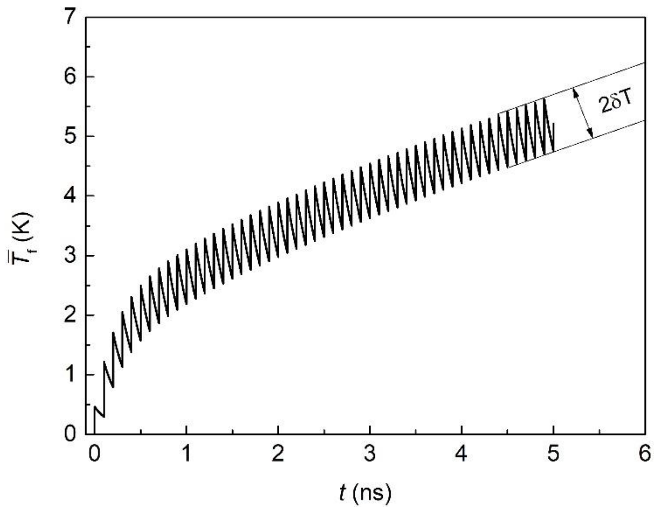

Supplementary Figure 1. The temporal evolution of the average temperature $\overline{T}_{\mathrm{f}}(t)$ for periodic laser pulse excitation with average power *W*=95 mW incident on the FeGa film with a thickness *h*=5nm deposited on the GaAs substrate.

$$\kappa_{\mathrm{f}}\frac{\partial T_{\mathrm{f}}}{\partial z}|_{z=0} = \kappa_{\mathrm{s}}\frac{\partial T_{\mathrm{s}}}{\partial z}|_{z=0} = (T_{\mathrm{f}} - T_{\mathrm{s}})/R, \quad (5)$$

where R is the thermal boundary resistance. At the interface of air and film, we assumed a zero heat flux boundary condition, i.e. $\kappa_{\mathrm{f}}\frac{\partial T_{\mathrm{f}}}{\partial z}|_{z=h} = 0$. We take the thickness of the substrate large enough to minimize the impact of any boundary condition at the back side of the substrate.

The described approach was implemented in COMSOL Multiphysics®. The example of the average lattice heating temperature of the film $\overline{T}_{\mathrm{f}} = \int_0^h \frac{T_{\mathrm{f}}dz}{h}$ for $W = 95$ mW, $r = 8.5$ µm is shown in Supplementary Figure 1. One can see that the temperature oscillates with the excitation frequency f_0=10 GHz around a slowly increasing background. Here we want to mention that our calculations show that in the discussed one-dimensional (1D) heat transport problem the background temperature does not reach a stationary value but, diverges with time. However, as one can clearly see from Supplementary Figure 1, the amplitude of thermal oscillations, δT, converges very quickly. In order to estimate the stationary background temperature ΔT we solved analytically the stationary 3D problem, see the next Supplementary Note.

Supplementary Note 2. Estimation of background temperature Δ*T*

Theory

In order to calculate the background temperature rise ΔT, we solved analytically the stationary 3D heat equation for T_{S}. Taking into account that the optical excitation energy is absorbed in a layer with a thickness much less than the radius of excitation at the surface, we may assume that the thermal source is located at the interface (*z*=0). Then the equation and boundary conditions may be written as:

$$\left(\frac{\partial^2}{\partial x^2} + \frac{\partial^2}{\partial y^2} + \frac{\partial^2}{\partial z^2}\right) T_{\mathrm{s}} = 0, \quad (6)$$

and

$$\kappa_{\mathrm{s}}\frac{\partial T_{\mathrm{s}}}{\partial z}|_{z=0} = (A_{\mathrm{f}} + A_{\mathrm{s}})\; I\; exp\left(-2\frac{x^2 + y^2}{r^2}\right), \quad (7)$$

where I is the laser intensity which is related to the laser power by

$$W = I \iint_{-\infty}^{\infty} exp\left(-2\frac{x^2+y^2}{r^2}\right) dxdy = I\frac{\pi r^2}{2}. \tag{8}$$

The analytical solution of these equations at *z*=0 and at the center of the laser beam (*x*=*y*=0) reads

$$\Delta T = \frac{(A_{\mathrm{f}} + A_{\mathrm{s}})W}{\sqrt{2\pi}\kappa_{s} r}. \tag{9}$$

It is obvious that for optical excitation from the side of the film (absorption length smaller in the metal than in the substrate) we have $T_{\mathrm{f}} > T_{\mathrm{s}}$ because of the boundary resistance. We estimate the jump of the temperature at the interface, using the boundary conditions, defined by Supplementary Equation 5. In this case, the temperature jump at the interface of the film and substrate is given by

$$(T_{\mathrm{f}} - T_{\mathrm{s}})|_{z=0} = (A_{\mathrm{f}} + A_{\mathrm{s}})IR = (A_{\mathrm{f}} + A_{\mathrm{s}})W\frac{2R}{\pi r^2}. \tag{10}$$

For the parameters of our experiment, the temperature ΔT (see Supplementary Equation 9) is 10 times larger than the temperature jump (see Supplementary Equation 10). Thus, the effect of the temperature jump can be neglected.

Experiment

We also derive the background temperature Δ*T* from the measured dependences of the resonance fields B_1, B_2, and B_3 on *W*. For this we take the derivatives *dB/dW* from the experimental data in Fig. 3a and compare them with the calculated derivatives *dB/dT* (see theoretical curves *f(B)* in Fig. 1(a) of the main text). As a result, we get the values for *dT/dW=(dB/dW)/(dB/dT)* for the three resonant frequencies 10, 20 and 30 GHz. The results are presented in the Supplementary Table 1.

Supplementary Table 1. The measured values of the resonant magnetic fields B_1, B_2, and B_3

Excitation Power (mW)	Resonant Fields (mT)		
	**10 GHz**	**20 GHz**	**30 GHz**
35	34	232	490
55	38	234	492
95	42	240	498

Supplementary Table 2. Power and temperature derivatives for the three resonant frequencies

	**10 GHz**	**20 GHz**	**30 GHz**
dB/dW **(mT mW^{-1})**	0.13	0.13	0.13
dB/dT **(mT K^{-1})**	0.19	0.13	0.16
dT/dW **(K mW^{-1})**	0.7	0.9	0.7

Supplementary Table 3. Values of the background temperature rise.

Power (mW)	ΔT (K)	
	**Calculated**	**Extracted from experiment**
35	18	28±2
55	28	44±3
95	48	77±5

The average value for dT/dW = 810±50 K W^{-1}. The estimated temperature rise ΔT= $(dT/dW)W$ for the three values of W are presented in Supplementary Table 3 and compared with the theoretical values. The difference between the values calculated and extracted from the experiments is less than 40%. This difference may be due to additional heating by the probe which is not included into the theoretical calculations.

Supplementary Note 3. Dependence of $\zeta=\delta T/\Delta T$ on parameters and design of the device

Supplementary Figure 2 shows the dependencies of the dynamical harvesting efficiency $\zeta=\delta T/\Delta T$ on the film thickness and thermal conductivity of the substrate. The value of ζ does not depend on power, because both δT and ΔT depend linearly on W. As one can see from Supplementary Figure 2a, ζ decreases with increasing film thickness. From Supplementary Figure 2b one can see that ζ increases linearly with thermal conductivity of the substrate, because ΔT is inversely proportional to κ_S (see Supplementary Equation 9).

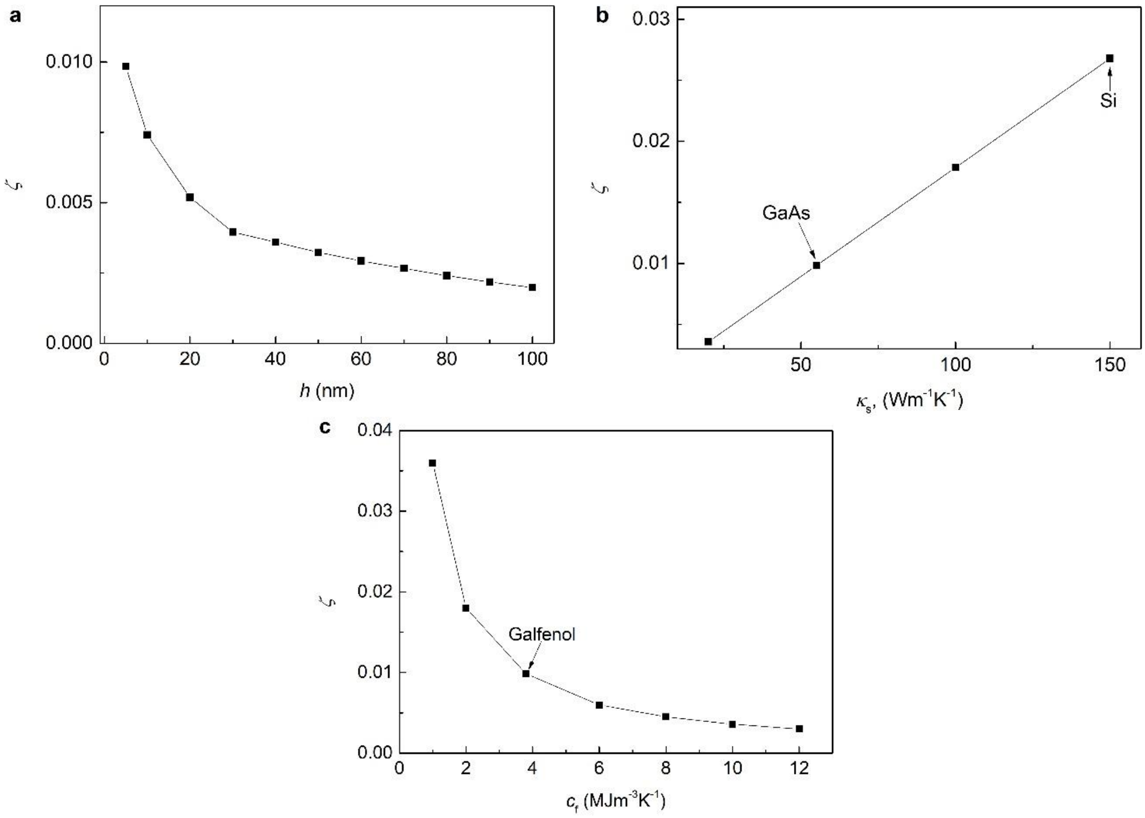

Supplementary Figure 2. Dependencies of the efficiency parameter, ζ on h (**a**), κ_S (**b**), and c_f (**c**).

Interestingly, in the considered frequency range, δT does not depend on R, κ_l, c_s, as well, and depends only on c_f. Thus, the parameter ζ is controlled by Supplementary Equation 9. Hence, in the case of a Si substrate, the efficiency can be more than two times larger than for GaAs, due to the difference in their heat conductivities. The dependence of the dynamical harvesting efficiency on c_f is shown in Supplementary Figure 2c. Thus, the efficiency can be larger than in Galfenol for materials with smaller c_f.

Finally, we discuss the design of a device with a ferromagnetic harvester, which uses the heat generated in a conducting layer in the vicinity of the harvester (see Supplementary Figure 3). Consider a thin conducting layer (e.g., a quantum well or a graphene layer) with thickness, l, which is separated from the harvester by a spacer with thickness, s. The harvester's thickness is h. We calculate ζ for such a device using an approach similar to that described in the Supplementary Notes 1 and 2. In our example, we take $h = l = 5$ nm, and $s = 2.5$ nm. For the conducting layer, spacer and substrate, we use the same thermal parameters as for GaAs. We consider that the heat is periodically released only in the conducting layer at f_0 =10 GHz. In the real device the heat source may be Joule heat emitted as result of an electrical current injected on the clock frequency f_0. For the harvester, we take Galfenol. In this case, the calculated efficiently ζ=0.004 which is only two times smaller than the efficiency of the system used in our experiments.

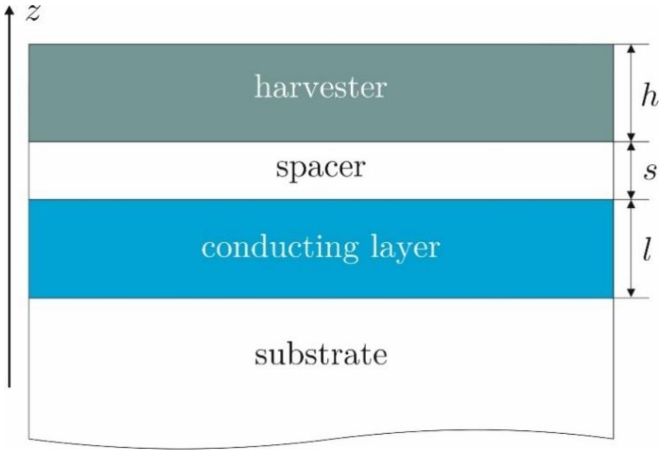

Supplementary Figure 3. Scheme of the device with a thin conducting layer and a ferromagnetic harvester.

Supplementary Note 4. Magnon dispersion

The frequency of thermally excited spin waves (magnons) in the studied Galfenol nanolayer is determined by the external magnetic field, **B**, and the magnon wave vector, **q**. The frequency of the ground magnon mode, which corresponds to the case of uniform precession (q=0), is given by the well-known expression [2]:

$$f = \frac{\gamma_0}{2\pi}\sqrt{F_{\phi\phi}F_{\theta\theta}}, \tag{11}$$

where γ_0 is the gyromagnetic ratio, and $F_{\theta\theta} = \frac{\partial^2 F_\mathbf{M}}{\partial\theta^2}$ and $F_{\phi\phi} = \frac{\partial^2 F_\mathbf{M}}{\partial\phi^2}$ are the second derivatives of the free energy density (see Eq. 2 in the main text) with respect to the in-plane azimuthal angle, ϕ, and polar angle, θ, calculated at the equilibrium orientation of magnetization. The angles are counted from the [100] crystallographic direction and from the normal to the layer

plane, respectively. Using the free energy density as in [3] and considering the case of an in-plane external magnetic field we get:

$$F_{\theta\theta} = B\cos(\phi_{\mathbf{M}} - \phi_{\mathbf{B}}) + \mu_0 M_0 + \frac{K_1}{2}(\cos4\phi_{\mathbf{M}} + 3) + K_{\mathrm{u}}(1 + sin2\phi_{\mathbf{M}}) \quad (12)$$

$$F_{\phi\phi} = B\cos(\phi_{\mathbf{M}} - \phi_{\mathbf{B}}) + 2K_1\cos4\phi_{\mathbf{M}} + 2K_{\mathrm{u}}\sin2\,\phi_{\mathbf{M}}$$

where K_1, K_{u} and M_0 are the temperature dependent cubic and uniaxial anisotropy coefficients, and the saturation magnetization, respectively; $\phi_{\mathbf{B}}$ is the in-plane angle of the external magnetic field and $\phi_{\mathbf{M}}$ is the field-dependent angle of the equilibrium orientation of magnetization (at B>0.3T, $\phi_{\mathbf{M}}=\phi_{\mathbf{B}}$). In the experimental temperature range the temperature dependences of K_1, K_u and M_0 may be written as: $X = X^{\mathrm{RT}} + \beta_X(T - T_0)$, where X= K_1, K_{u} or M_0 [4]; X^{RT} are their values at room temperature and $\beta_X = \frac{\partial X}{\partial T}$ is the temperature independent coefficient. The dependences $f(B)$ calculated for $\phi_{\mathbf{B}}=-\pi/8$ for room temperature and ΔT=200 K are shown in Fig. 1a of the main text. The parameters used for the calculations are the following [3,4]: $M_0^{\mathrm{RT}} = 1.95$ T, $K_1^{\mathrm{RT}} = 20$ mT, $K_{\mathrm{u}}^{\mathrm{RT}} = 9$ mT, $\beta_{\mathbf{M}} = -0.97$ mT K^{-1}, $\beta_{K_1} = -\,0.046$ mT K^{-1}, $\beta_{K_{\mathrm{u}}} = -0.025$ mT K^{-1}.

Due to the finite laser spot size, the thermal modulation generates also magnons with non-zero in-plane wave vectors, $q_{||}$. The range of in-plane wave vectors is set by the laser spot. It is found from the Fourier transform of the Gaussian distribution of the laser intensity (see Supplementary Equation 7). Therefore, we obtain $0 \le q_{||} \le \frac{4}{r}$, where r is the laser spot radius. In our experiment with r = 8.5 μm the upper limit is $q_{||} \le 4700$ cm^{-1}. This range corresponds to magneto-static spin waves with a very weak dispersion determined by the magnetic dipole-dipole interaction. This dispersion is strongest for the wave vectors perpendicular to the external magnetic field [5]. In the case of a thin ferromagnetic film of thickness, h, where $\frac{q_{||}h}{2\pi}$<<1 it can be written in a simplified form as [6]:

$$f_{q_{||}} = f + \frac{\gamma_0}{2\pi}\nu\, q_{||}h, \quad (13)$$

where ν is the dispersion coefficient determined by the main parameters of the ferromagnet: K_1, K_{u} and M_0. For the studied Galfenol layer its value at room temperature is $\nu \approx 3.8$ T [7] and in our experiment the frequencies of the thermally driven magnons with finite in-plane wave vector do not exceed 270 MHz. This value is less than the spectral width of the fundamental magnon mode (0.5 GHz) determined by the precession decay. Thus, the thermally driven magnons with finite in-plane wave vectors may be considered as degenerate.

The spectrum of magnons with finite tangential wave vector, $q_\perp$, is quantized due to spatial confinement along the layer normal: $q_{\perp,n} = \frac{\pi n}{h}$, where n=1,2,3… In layers with nanometer thickness, the corresponding dispersion is determined by the exchange interaction and in a simplified form can be written as:

$$f_n = f + \frac{\gamma_0}{2\pi}\varrho D q_{\perp,n}^2, \quad (14)$$

where D is the spin stiffness constant and ϱ is a field dependent coefficient approaching 1 with increasing B [8]. In the studied Galfenol layer with h=5 nm, the large penetration depth of the laser pulse in comparison with the layer thickness results in uniform thermal distribution along the layer normal. Thus, the odd magnon modes (n=1,3,5) cannot be excited. The lowest higher-order mode, which can be driven by thermal modulation, corresponds to n=2. For the Galfenol spin stiffness D=1.5×10^{-17} Tm^2 [8, 9], the frequency of this mode exceeds the ground mode

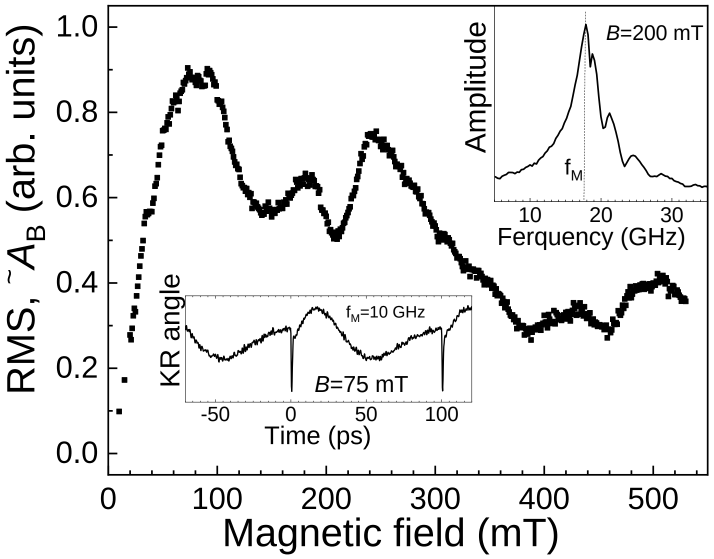

Supplementary Figure 4. Magnetic field dependences of the root mean square (RMS) Kerr rotation amplitude, $\widetilde{A_B}$, measured in a Galfenol layer of 105-nm thickness for the excitation power *W*=140 mW. The upper inset shows the magnon spectrum obtained as the fast Fourier transform of the Kerr rotation signal measured in a single pulse pump-probe experiment at *B*=200 mT. The lower inset shows the transient Kerr rotation signal measured for 10 GHz optical excitation at *B*=75 mT.

frequency by more than 500 GHz. Due to the extremely low amplitude of such high-frequency harmonics in the thermal spectrum, this mode as well as other higher-order even modes are not excited. Thus, in the studied layer of 5-nm thickness the fundamental magnon mode is solely driven by the modulated heat.

The situation changes drastically in a ferromagnetic layer with thickness ~100 nm, which possesses significantly less spectral splitting of the higher-order magnon modes with finite $q_\perp$. Due to the small penetration depth of light in comparison with the layer thickness, both odd and even modes can be excited [8,10]. This results in a drastic broadening of the magnon spectrum and a corresponding dephasing of the magnetization precession with destructive effects on the resonant energy harvesting. This is clearly seen in Supplementary Figure 4, which summarizes the experimental results obtained for a Galfenol layer of thickness *h*=105 nm. The upper inset shows the magnon spectrum obtained by fast Fourier transforming the Kerr rotation signal measured in a single pulse pump-probe experiment at *B*=200 mT. In addition to the dominating fundamental magnon mode, the FFT includes several spectral lines, which correspond to the higher-order magnon modes with *n*=1…5. Their spectral splitting is described with high accuracy by the dispersion relation [7]. The result of such a spectral broadening becomes apparent in the transient KR signal measured under resonant conditions when $f=f_0$=10 GHz (see the lower inset). The non-harmonic character of the thermally driven oscillations in the KR signal is clearly seen. As a result, the field dependence of the RMS Kerr amplitude shown in the main panel consists of several broad peaks instead of narrow resonances as observed in the 5-nm Galfenol layer. At *B*=75 mT, which corresponds to the maximum amplitude of precession, its absolute values and RMS are 15-times smaller than in the 5-nm layer.

Supplementary References

[1] COMSOL Multiphysics® v. 5.4. http://www.comsol.com. COMSOL AB, Stockholm, Sweden.

[2] Gurevich, A.G. & Melkov, G.A., Magnetization oscillations and waves (CRC-Press, Boca Raton, 1996).

[3] Kats, V. N. et al., Ultrafast changes of magnetic anisotropy driven by laser-generated coherent and noncoherent phonons in metallic films, *Phys. Rev. B* **93**, 214422 (2016).

[4] Clark, A. E. et al., Temperature dependence of the magnetic anisotropy and magnetostriction of $Fe_{100-x}Ga_x$ (x=8.6, 16.6, 28.5). *J. of Appl. Phys.* 97, 10M316 (2005).

[5] Damon, R. W. & ESHBACH J. R., *J. Phys. Chem. Solids* **19**, 308-320 (1961).

[6] Kamimaki, A., Iihama, S., Sasaki, Y., Ando, Y., & Mizukami, S. Reciprocal excitation of propagating spin waves by a laser pulse and their reciprocal mapping in magnetic metal films. *Phys. Rev. B* **96**, 014438 (2017).

[7] Khokhlov, N. E. et al., Optical excitation of propagating magnetostatic waves in an epitaxial galfenol film by ultrafast magnetic anisotropy change. Phys. Rev. Appl. **12**, 044044 (2019).

[8] Scherbakov, A.V. et al., Optical excitation of single- and multimode magnetization precession in Fe-Ga nanolayers. *Phys. Rev. Appl.* **11**, 031003 (2019).

[9] Gopman, D. B., Sampath, V., Ahmad, B. H., Bandyopadhyay, S., & Atulasimha, J., Static and dynamic magnetic properties of sputtered Fe-Ga thin films, *IEEE Trans Magn.* **53**, 6101304 (2017).

[10] van Kampen, M. et al., All-optical probe of coherent spin waves. *Phys. Rev. Lett.* **88**, 227201 (2002).
